# Association between the atherogenic index of plasma and abdominal aortic calcification: results from the National Health and Nutrition Examination Survey 2013–2014

**DOI:** 10.3389/fendo.2025.1472267

**Published:** 2025-02-17

**Authors:** Xiaozhou Su, Chunli Zhao, Donghua Li, Xianwei Zhang

**Affiliations:** Department of Cardiology, Minzu Affiliated Hospital of Guangxi Medical University, Nanning, Guangxi, China

**Keywords:** atherogenic index of plasma, abdominal aortic calcification, atherosclerosis, cross-sectional study, NHANES

## Abstract

**Background:**

Coronary artery calcification and cardiovascular disease are associated with elevated levels of atherogenic plasma index (AIP). However, the relationship with abdominal aortic calcification (AAC) remains unclear. This study aimed to explore the association between AIP and AAC using the National Health and Nutrition Examination Survey (NHANES) database.

**Methods:**

A cross-sectional analysis was conducted on 2,811 individuals aged 40 years or older from the 2013–2014 NHANES dataset. Participants with missing AAC-24 scores, AIP data, or covariate information were excluded. AAC was quantified using the Kauppila score (AAC-24), with a score > 0 indicating the presence of AAC, and severe AAC (SAAC) being defined as an AAC-24 score ≥ 6. Multivariable regression models and restricted cubic spline analyses were employed to assess the associations between AIP and AAC. Sensitivity analysis was used to validate the robustness of the findings.

**Results:**

The study population had a mean age of 57.7 years, with 48.22% being male. A significant positive association was found between AIP and both the AAC score and the risk of AAC and SAAC, particularly in females. For the overall population, each unit increase in AIP was associated with an overall increase in AAC-24 score of 0.90 (95% CI: 0.22, 1.58; p = 0.009), and for women, the AAC risk and SAAC risk would be 4.01-fold higher (95% CI: 1.65, 9.74; p = 0.002) and 9.37-fold higher (95% CI: 2.37, 37.03; p = 0.001). No significant associations were found in males. Further analysis revealed a significant interaction between AIP and gender regarding both AAC scores and the risk of SAAC.

**Conclusions:**

This study demonstrates a positive relationship between AIP and increased AAC scores, as well as a higher risk of AAC and SAAC in U.S. women. However, these findings require further investigation to confirm the observed gender-specific differences.

## 1 Introduction

Cardiovascular diseases (CVDs), the primary cause of noncommunicable diseases globally, claimed 12 million lives in 1990 and 18.6 million in 2019 (1). Projections indicate that by 2030, over 40% of the U.S. population will be affected (2). Atherosclerosis, a key driver of CVD, underscores the importance of early detection and intervention to mitigate adverse outcomes. Atherosclerosis underlies many CVDs, and abdominal aortic calcification (AAC) is a well-established marker of subclinical atherosclerosis. AAC is independently associated with coronary artery disease (CAD) (3), stroke (4), and peripheral artery disease (5). Thus, early detection and a better understanding of the risk factors associated with AAC are crucial for effective cardiovascular risk assessment and management.

In 2001, Dobiásová and Fröhlich introduced the atherogenic plasma index (AIP), calculated as the logarithm of the molar ratio of triglycerides to high-density lipoprotein cholesterol (TG/HDL-C) (6). AIP is a reliable indicator of dyslipidemia and atherosclerosis, surpassing traditional lipid markers in predicting CVDs and metabolic disturbances (7), such as metabolic syndrome (8), hypertension (9), and the risk of CVDs and associated mortality (10, 11). Recent studies have also highlighted a J-shaped relationship between AIP and cardiovascular outcomes, including myocardial infarction in hypertensive patients with obstructive sleep apnea (12). When compared with other cardiovascular risk markers, such as triglycerides and total cholesterol, AIP has demonstrated superior predictive power in certain populations. A meta-analysis indicated a stronger correlation between AIP and type 2 diabetes mellitus (T2DM) than with other traditional lipid markers (13). Furthermore, another study found a more significant association between AIP and in-stent restenosis in patients with acute coronary syndrome, particularly among those with LDL-C levels greater than 1.8 mmol/L (14). These studies suggest that AIP is particularly effective in identifying individuals with metabolic disturbances, such as insulin resistance (IR) or dyslipidemia, which may not be fully captured by standard lipid measurements. This highlights the broader relevance of AIP as a cardiovascular risk marker, a perspective our study seeks to expand by exploring its association with AAC. Given its simplicity and ability to accurately reflect atherosclerotic lipid profiles, AIP has the potential to serve as a valuable tool for early AAC screening and, when combined with other cardiovascular risk factors, to aid in comprehensive cardiovascular risk assessment.

Sex differences play a significant role in cardiovascular health. Men and women exhibit significant differences in metabolism and hormone levels, which influence lipid metabolism (15), vascular function (16), and the development of atherosclerosis (17). Estrogen, a key factor driving these differences, provides cardioprotective effects in premenopausal women by improving lipid profiles (increasing HDL-C and lowering LDL-C), reducing inflammation, and inhibiting vascular calcification (18). However, postmenopausal women experience hormonal changes that can alter their lipid profile, including elevated LDL-C, decreased HDL-C, and higher triglycerides. These changes may also amplify AIP levels, potentially strengthening the association between AIP and AAC. Some studies have noted a stronger association between AIP and conditions like hyperuricemia (19) and CAD (20) in women than in men. This suggests that more targeted screening and preventive strategies may be needed. These hormonal changes may explain why postmenopausal women, despite similar traditional risk factors, are at greater risk for cardiovascular disease, particularly accelerated AAC (21). Identifying the sex-specific relationship between AIP and AAC can help tailor more effective treatment plans for specific populations, ultimately reducing the long-term cardiovascular burden in high-risk groups.

Although the clinical significance of sex differences has garnered increasing attention, research on the relationship between AIP and AAC, especially considering sex-specific effects, remains limited. Therefore, the aim of this study is to fill this gap by exploring the relationship between AIP and AAC, with a focus on the potential role of sex in this association. We hypothesize that the association between AIP and AAC varies by gender, potentially due to differences in lipid metabolism and hormonal regulation. From a public health perspective, sex-specific screening, routine AIP monitoring, and timely interventions could enhance early CVD detection and enable precision prevention, with AIP serving as a critical biomarker, particularly in postmenopausal women.

## 2 Materials and methods

### 2.1 Study design and participants

**Figure 1:**
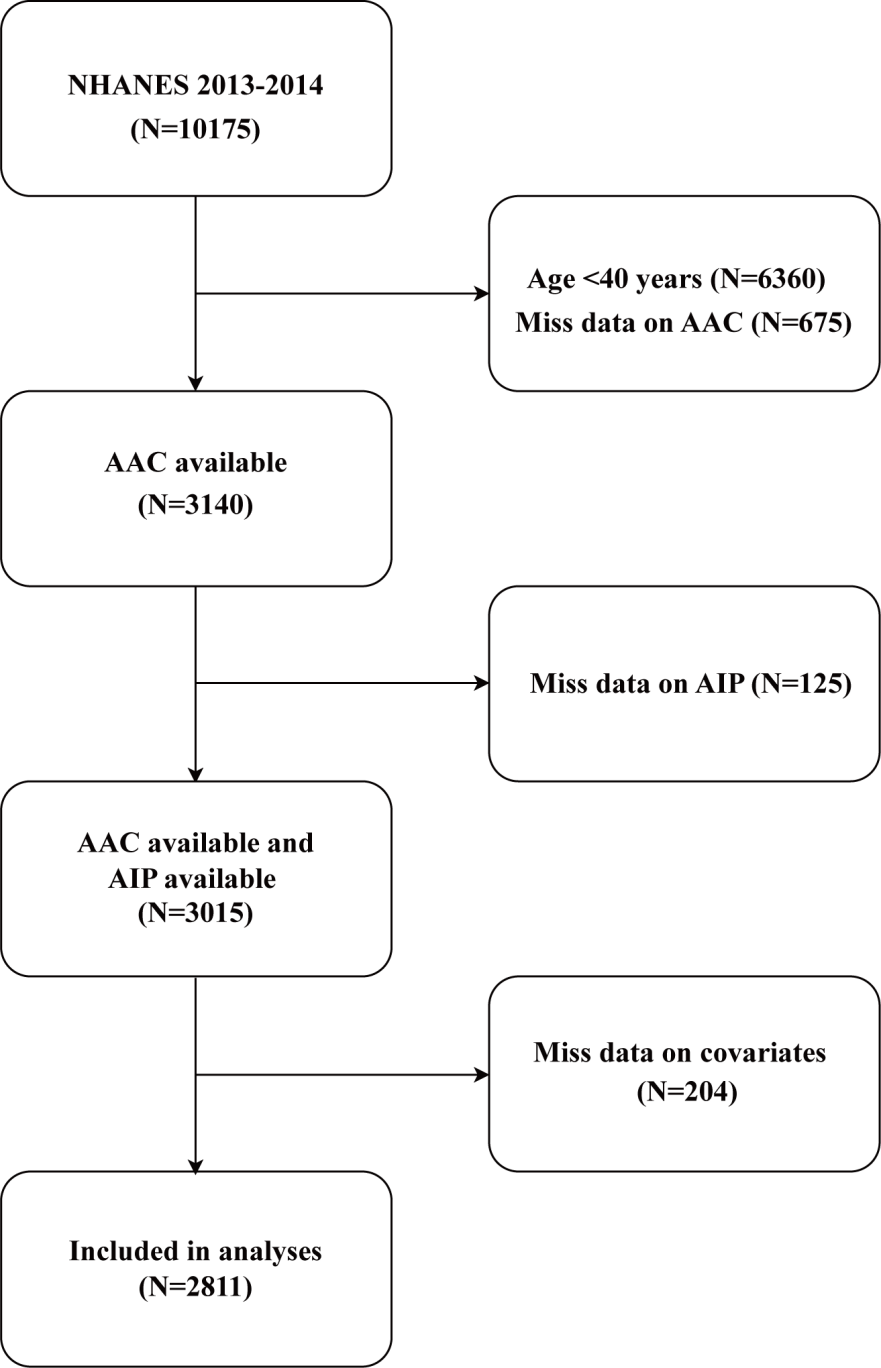
Flow chart of participant selection. NHANES, National Health and Nutrition Examination Survey; AIP, atherogenic index of plasma; AAC, abdominal aortic calcification.

This study utilized data from the NHANES (https://www.cdc.gov/nchs/nhanes/). NHANES employs a multistage probability sampling design and integrates interviews, physical examinations, and laboratory tests to generate comprehensive and representative health and nutrition data. All analyses adhered to NHANES-recommended protocols, incorporating sampling weights to account for the survey’s complex design and mitigate non-response bias. Rigorous quality control measures were implemented throughout the survey process to ensure data accuracy and reliability. The initial sample included 10,175 participants. After applying the inclusion and exclusion criteria, the final analysis sample consisted of 2,811 participants aged 40 years or older (**Figure 1**). Participants were excluded for the following reasons: 6,360 individuals were under the age of 40, as AAC-24 data are only available for participants aged 40 years or older, ensuring consistency with NHANES data availability. Additionally, 675 participants were excluded due to missing or incomplete AAC-24 scores, while 125 individuals were excluded due to the absence of AIP data. Lastly, 204 participants were excluded because of missing information on key covariates, including education level, marital status, smoking status, alcohol drinking status, hypertension, high cholesterol, or diabetes status. While excluding participants with incomplete data may introduce selection bias and limit generalizability, sensitivity analyses using multiple imputation (MI) were performed to evaluate the impact of missing data and ensure the robustness of the findings.

The NHANES study protocol was approved by the Ethics Review Board of the National Center for Health Statistics (NCHS). All participants provided written informed consent before enrollment.

### 2.2 Definitions of AAC and AIP

The primary exposure variable, AIP, was calculated from blood samples using the formula log10 (triglycerides/HDL-C) (22). Participants were stratified into quartiles based on AIP distribution: Q1 (-0.682 to -0.153), Q2 (0.154 to 0.392), Q3 (0.393 to 0.650), and Q4 (0.651 to 2.169). Quartiles were determined using a data-driven statistical approach by ranking AIP values from lowest to highest and dividing them into four equal-sized groups. This approach minimizes bias from arbitrary cutoffs and is widely applied in epidemiological research (23), enhancing the robustness of the analysis and facilitating the examination of associations between AIP and AAC risk. AAC was assessed using lateral spine images obtained via dual-energy X-ray absorptiometry (DXA, Densitometer Discovery A, Hologic, Marlborough, MA, USA) and lateral spine vertebral scans conducted at NHANES mobile examination centers. AAC was scored using the AAC-24 and AAC-8 methods (Kauppila score) (24). An AAC-24 score >0 was used to define AAC, while severe AAC (SAAC) was defined as AAC-24 ≥6, consistent with thresholds established in previous studies (25, 26). The cutoff was selected due to its clinical relevance and its strong association with elevated cardiovascular risk, as reported in prior studies (27). Furthermore, sensitivity analysis included the AAC-8 score and severe AAC (AAC-8 ≥ 3) (28, 29). Details of AAC measurement protocols are available at https://wwwn.cdc.gov/Nchs/Nhanes/2013-2014/DXXAAC_H.htm.

### 2.3 Covariates

Potential confounders were identified through a literature review and included demographic variables (age, gender, race, degree of education, marital status, and poverty income ratio (PIR)), lifestyle factors (such as drinking alcohol and smoking), physical examination data (waist circumference, body mass index (BMI), systolic blood pressure (SBP), diastolic blood pressure (DBP)), biochemical markers (total cholesterol (TC), HDL-C, triglycerides (TG), total protein, albumin, aspartate aminotransferase (AST), alanine aminotransferase (ALT), serum calcium, serum phosphorus, serum creatinine, estimated glomerular filtration rate (eGFR), total bilirubin, serum uric acid, total 25-hydroxyvitamin D), and comorbidities (hypertension, high cholesterol, diabetes). Standardized household questionnaires were used to collect demographic data and traits. The categories for race and ethnicity were Other Hispanic, Mexican American, Non-Hispanic White, Non-Hispanic Black, and Other. Education level was classified as below high school, high school or equivalent, or college or above. Unmarried and married were the two categories for marital status. Drinkers were defined as those who claimed to have had at least 12 alcoholic drinks in a given year. If an individual had smoked 100 cigarettes or more during their lifetime, they were considered smokers. Having a medical diagnosis of hypertension, taking medication for hypertension, or having an average systolic blood pressure of at least 140 mmHg and/or a diastolic blood pressure of at least 90 mmHg were all considered to be indicators of hypertension (30). TC ≥ 240 mg/dL during fasting or while using lipid-lowering medications was considered high cholesterol (31). The criteria for diabetes were hemoglobin A1c value ≥ 6.5%, a fasting plasma glucose value ≥ 126 mg/dL, or a 2-hour plasma glucose value ≥ 200 mg/dL, in addition to a diagnosis of diabetes or the use of hypoglycemic medications (32). Four continuous blood pressure readings were taken. The average of four blood pressure readings was used to define SBP and DBP in this study. Every participant was required to supply an immediate venous blood sample overnight. Body mass index (BMI) was calculated as weight (kg) divided by height squared (m). The Chronic Kidney Disease Epidemiology Collaboration (CKD-EPI) equation was the method used to determine the glomerular filtration rate (eGFR) (33). The study variables’ comprehensive measurement procedures are accessible to the general public at www.cdc.gov/nchs/nhanes/.

### 2.4 Statistical analysis

Following the NHANES analysis guidelines, survey-weighted means (95% CIs) were used to describe continuous variables, while survey-weighted percentages with 95% CIs were used for categorical variables. AIP was analyzed both as a continuous variable and as a categorical variable (quartiles, with the first quartile serving as the reference). Group differences were assessed using weighted chi-square tests for categorical variables and weighted logistic regression models for continuous variables. The relationship between AIP and AAC was examined using multivariable logistic regression models. The selection of potential covariates for the multivariable regression models was based on the following criteria: (1) relevant demographic characteristics, including factors such as age, gender, and race, which have been shown to influence both AIP and AAC in prior studies; (2) variables shown to affect AIP and/or AAC in previous studies (34, 35), ensuring that known cardiovascular risk factors were considered in the models; (3) variables whose inclusion resulted in a change of more than 10% in the coefficients of the basic model, in accordance with the STROBE statement (36), the basic model changes by more than 10% after the introduction of covariates; (4) other variables based on clinical experience, including factors that could influence the outcomes but were not captured in the previous categories. The selection process aimed to control for confounding by incorporating these factors, ensuring that the estimated relationship between AIP and AAC was not biased by external influences. Furthermore, model validation was conducted through sensitivity analyses to ensure robustness. Collinearity among independent variables was assessed using variance inflation factors (VIFs), with a VIF > 10 indicating significant multicollinearity that could compromise regression stability. Model 1 represented the unadjusted data. In Model 2, the data were adjusted for age, gender, and race. In Model 3, results were adjusted for age, gender, race, education level, marital status, smoking status, alcohol drinking status, waist circumference, BMI, PIR, total cholesterol, triglycerides, hemoglobin A1c, albumin, total bilirubin, AST, ALT, blood urea nitrogen, serum creatinine, eGFR, serum calcium, serum phosphorus, total protein, serum uric acid, total 25-hydroxyvitamin D, hypertension, high cholesterol, and diabetes status. To assess potential non-linear relationships between AIP and AAC, restricted cubic spline (RCS) analyses were conducted with four knots placed at 0.15, 0.35, 0.65, and 0.95. These placements were based on data-driven criteria and prior recommendations to capture non-linear trends while minimizing overfitting (37). Non-linearity was evaluated using the Wald test. A P-value for non-linearity < 0.05 was considered evidence of a non-linear association, whereas a P-value ≥ 0.05 indicated the absence of significant non-linearity.

Furthermore, stratified analyses were conducted to evaluate effect modification by variables such as gender, age, BMI, eGFR, alcohol drinking status, smoking status, and the presence of hypertension and diabetes. All covariates were adjusted except for the stratification variable itself. Interaction tests were performed by including interaction terms between AIP and the stratification variables in the regression models. Interaction p-values were reported to assess subgroup differences.

To validate the findings, sensitivity analyses were conducted using the AAC-8 scoring system and MI methods. Covariates with missing values included education level, marital status, smoking status, alcohol drinking status, hypertension, high cholesterol, or diabetes status. MI with chained equations was used to address missing data, with five imputations performed for each missing value. The imputation model included all covariates and variables potentially predicting missingness. Results from complete-case analyses were compared with those obtained from the imputed dataset to evaluate the impact of missing data on study conclusions. All statistical analyses were performed using R (version 4.2.0, R Foundation) and EmpowerStats (X&Y Solutions, Inc., Boston, MA). A two-sided p-value < 0.05 was considered statistically significant.

## 3 Results

### 3.1 Baseline characteristics of participants

The weighted baseline characteristics of all participants are presented in **Table 1**. The mean age of the participants was 57.74 ± 11.51 years, with 48.22% being male. The overall mean AIP was 0.40 ± 0.36, and the quartile ranges were as follows: Q1 (-0.682, -0.153), Q2 (0.154, 0.392), Q3 (0.393, 0.650), and Q4 (0.651, 2.169). Notably, participants in the highest AIP quartile (Q4) were more likely to be male and of Mexican American ethnicity and had lower education levels compared to those in lower quartiles (P < 0.05). Q4 participants also exhibited higher rates of hypertension, diabetes, and hypercholesterolemia (all P < 0.05), indicating a greater burden of cardiovascular risk factors. Q4 participants exhibited significantly higher levels of multiple metabolic markers, including waist circumference, BMI, TG, HbA1c, ALT, and serum creatinine (all P < 0.05). These abnormalities, closely associated with arterial calcification, may illuminate potential mechanisms linking higher AIP to AAC. Furthermore, participants in the Q4 group had significantly higher levels of total 25-hydroxyvitamin D, which may reflect metabolic dysfunction and an increased risk of AAC in this group. Overall, individuals with higher AIP not only exhibited higher cardiovascular risk factors but also had a higher prevalence of AAC and higher AAC-24 scores, further supporting the potential role of AIP as a marker for AAC risk.

**Table 1:** Weighted baseline characteristics of participants by quartiles of baseline AIP.

Variable^a^	Overall	AIP Quartiles				P-value
		Q1 (-0.682-0.153)	Q2 (0.154-0.392)	Q3 (0.393-0.650)	Q4 (0.651-2.169)	
Participants	2811	703	702	703	703	
Age (year)	57.74 (57.17,58.32)	57.44 (56.35,58.53)	57.79 (56.58,58.99)	58.55 (57.45,59.65)	57.25 (56.09,58.41)	0.572
Gender (%)						<0.001
Male	48.22 (46.29,50.14)	34.26 (30.18,38.58)	44.55 (41.33,47.83)	53.73 (47.74,59.61)	60.97 (56.97,64.83)	
Female	51.78 (49.86,53.71)	65.74 (61.42,69.82)	55.45 (52.17,58.67)	46.27 (40.39,52.26)	39.03 (35.17,43.03)	
Race (%)						<0.001
Mexican American	6.80 (4.08,11.13)	3.76 (2.08,6.70)	6.08 (3.24,11.15)	8.69 (5.47,13.55)	8.85 (5.38,14.21)	
Other hispanic	4.59 (3.07,6.79)	3.39 (2.12,5.36)	4.44 (2.73,7.16)	5.28 (3.58,7.73)	5.30 (3.48,7.99)	
Non-Hispanic White	72.28 (65.33,78.30)	73.21 (67.19,78.48)	71.95 (62.78,79.59)	70.68 (63.24,77.16)	73.16 (65.46,79.67)	
Non-Hispanic Black	9.68 (7.31,12.71)	14.35 (11.04,18.44)	11.07 (7.84,15.41)	8.20 (5.91,11.29)	4.90 (3.37,7.06)	
Other Race	6.66 (5.16,8.55)	5.30 (3.67,7.59)	6.46 (4.28,9.63)	7.14 (5.51,9.21)	7.80 (5.21,11.52)	
Education level (%)						<0.001
Less than high school	4.63 (3.34,6.38)	2.95 (1.82,4.77)	4.16 (2.65,6.48)	6.49 (4.55,9.17)	5.04 (3.01,8.32)	
High school or equivalent	31.86 (27.16,36.96)	28.10 (22.58,34.38)	28.06 (23.19,33.51)	31.19 (26.77,35.97)	40.07 (31.09,49.77)	
College or above	63.51 (57.40,69.22)	68.94 (62.11,75.04)	67.78 (61.02,73.87)	62.32 (56.02,68.23)	54.89 (46.07,63.41)	
Marital status (%)						0.374
Married	66.02 (63.08,68.85)	64.34 (57.79,70.40)	63.85 (57.64,69.63)	66.82 (61.15,72.04)	69.12 (66.32,71.78)	
Unmarried	33.98 (31.15,36.92)	35.66 (29.60,42.21)	36.15 (30.37,42.36)	33.18 (27.96,38.85)	30.88 (28.22,33.68)	
Smoker (%)						<0.001
Yes	45.62 (41.86,49.43)	38.82 (33.15,44.82)	42.22 (36.06,48.62)	51.33 (46.64,56.00)	50.54 (45.42,55.64)	
No	54.38 (50.57,58.14)	61.18 (55.18,66.85)	57.78 (51.38,63.94)	48.67 (44.00,53.36)	49.46 (44.36,54.58)	
Alcohol user (%)						0.578
Yes	78.13 (73.96,81.80)	79.76 (74.92,83.87)	77.29 (69.52,83.55)	78.81 (74.53,82.55)	76.62 (71.79,80.84)	
No	21.87 (18.20,26.04)	20.24 (16.13,25.08)	22.71 (16.45,30.48)	21.19 (17.45,25.47)	23.38 (19.16,28.21)	
PIR	3.15 (2.92, 3.37)	3.38 (3.13,3.62)	3.29 (3.06,3.53)	2.93 (2.69,3.17)	2.98 (2.72,3.23)	<0.001
Waist circumference (cm)	100.01 (99.37 100.65)	92.33 (90.93,93.73)	98.08 (96.73,99.42)	103.65 (102.58,104.72)	106.40 (105.16,107.64)	<0.001
BMI (kg/m^2^)	28.58 (28.27, 28.89)	26.04 (25.49,26.58)	27.97 (27.44,28.49)	29.90 (29.43,30.38)	30.55 (29.88,31.21)	<0.001
SBP (mmHg)	125.38 (124.30, 126.45)	123.16 (121.10,125.22)	124.64 (123.10,126.17)	126.22 (124.85,127.59)	127.58 (125.37,129.79)	0.122
DBP (mmHg)	70.68 (69.83, 71.53)	69.23 (68.41,70.04)	70.46 (69.59,71.33)	70.50 (68.96,72.04)	72.55 (71.17,73.93)	0.001
HDL-C (mg/dL)	54.84 (54.15, 55.54)	73.01 (71.48,74.53)	57.52 (56.48,58.56)	48.18 (47.27,49.09)	39.77 (39.00,40.54)	<0.001
TG (mg/dL)	160.11 (153.77, 166.44)	67.95 (66.53,69.37)	108.86 (106.19,111.53)	157.67 (153.06,162.28)	307.37 (289.89,324.85)	<0.001
TC (mg/dL)	195.48 (194.46, 196.50)	190.84 (187.44,194.25)	193.01 (189.10,196.91)	193.90 (189.91,197.89)	204.18 (200.60,207.76)	0.010
HbA1c (%)	5.78, (5.73, 5.82)	5.51 (5.42,5.59)	5.61 (5.54,5.68)	5.88 (5.79,5.97)	6.12 (5.97,6.27)	<0.001
Total protein (g/dL)	6.96 (6.91, 7.00)	6.91 (6.86,6.95)	6.91 (6.87,6.95)	7.02 (6.95,7.09)	7.00 (6.95,7.05)	0.001
Albumin (g/dL)	4.25 (4.23, 4.27)	4.25 (4.22,4.28)	4.25 (4.22,4.27)	4.24 (4.19,4.28)	4.26 (4.24,4.29)	0.798
AST (U/L)	25.40 (24.35, 26.46)	24.71 (23.90,25.53)	24.54 (23.15,25.93)	25.50 (24.28,26.72)	26.86 (24.36,29.36)	0.209
ALT (U/L)	24.57 (23.40, 25.74)	21.18 (20.36,22.01)	23.74 (22.04,25.44)	25.72 (23.89,27.55)	27.78 (26.13,29.43)	<0.001
BUN (mg/dL)	14.27 (14.05, 14.50)	13.85 (13.36,14.35)	14.15 (13.61,14.69)	14.67 (14.23,15.11)	14.45 (14.00,14.89)	0.063
Serum calcium (mg/dL)	9.45 (9.43, 9.48)	9.44 (9.42,9.47)	9.42 (9.38,9.47)	9.47 (9.43,9.51)	9.48 (9.45,9.52)	0.120
Serum phosphorus (mg/dL)	3.80 (3.76, 3.83)	3.83 (3.77,3.89)	3.78 (3.73,3.82)	3.78 (3.72,3.84)	3.81 (3.74,3.87)	0.545
Serum creatinine (mg/dL)	0.93 (0.91, 0.95)	0.89 (0.87,0.92)	0.92 (0.89,0.95)	0.94 (0.91,0.97)	0.97 (0.94,0.99)	0.001
eGFR (mL/min/1.73 m^2^)	82.70 (81.67, 83.72)	83.77 (82.45,85.09)	82.56 (80.91,84.21)	82.32 (80.37,84.26)	82.08 (80.35,83.82)	0.143
Total Bilirubin (mg/dL)	0.65 (0.63, 0.67)	0.70 (0.67,0.72)	0.67 (0.63,0.71)	0.61 (0.58,0.64)	0.63 (0.60,0.65)	0.001
Serum uric acid (mg/dL)	5.42 (5.35, 5.49)	4.86 (4.78,4.94)	5.28 (5.21,5.36)	5.64 (5.52,5.76)	5.92 (5.72,6.12)	<0.001
Total 25-hydroxyvitamin D (nmol/L)	75.40 (72.66, 78.14)	80.38 (76.18,84.57)	76.38 (72.47,80.29)	74.04 (70.80,77.28)	70.59 (66.96,74.23)	0.001
Hypertension (%)						<0.001
Yes	50.89 (48.39,53.38)	41.73 (35.64,48.10)	47.72 (42.13,53.37)	55.52 (51.34,59.63)	59.03 (54.16,63.73)	
No	49.11 (46.62,51.61)	58.27 (51.90,64.36)	52.28 (46.63,57.87)	44.48 (40.37,48.66)	40.97 (36.27,45.84)	
High cholesterol (%)						<0.001
Yes	56.61 (54.50,58.68)	41.88 (37.46,46.43)	53.21 (46.09,60.21)	60.29 (55.61,64.78)	71.63 (68.37,74.67)	
No	43.39 (41.32,45.50)	58.12 (53.57,62.54)	46.79 (39.79,53.91)	39.71 (35.22,44.39)	28.37 (25.33,31.63)	
Diabetes (%)						<0.001
Yes	17.16 (15.54,18.90)	8.11 (5.60,11.60)	12.45 (9.61,15.98)	20.50 (15.60,26.46)	27.91 (23.66,32.60)	
No	82.84 (81.10,84.46)	91.89 (88.40,94.40)	87.55 (84.02,90.39)	79.50 (73.54,84.40)	72.09 (67.40,76.34)	
AAC24 score	1.48 (1.28, 1.69)	1.15 (0.77,1.53)	1.45 (1.24,1.66)	1.70 (1.36,2.04)	1.65 (1.29,2.01)	0.008
AAC (%)						0.001
Yes	29.17 (25.51,33.13)	22.82 (16.53,30.62)	29.48 (25.95,33.26)	34.12 (30.43,38.02)	30.76 (25.34,36.77)	
No	70.83 (66.87,74.49)	77.18 (69.38,83.47)	70.52 (66.74,74.05)	65.88 (61.98,69.57)	69.24 (63.23,74.66)	
SAAC (%)						0.215
Yes	9.66 (8.30,11.20)	7.44 (5.36,10.25)	9.65 (7.88,11.76)	10.92 (7.78,15.11)	10.75 (7.88,14.51)	
No	90.34 (88.80,91.70)	92.56 (89.75,94.64)	90.35 (88.24,92.12)	89.08 (84.89,92.22)	89.25 (85.49,92.12)	

a Data were summarized as mean ± SD or frequency (percentage) according to their data type.PIR, Poverty income ratio; BMI, body mass index; SBP, systolic blood pressure; DBP, diastolic blood pressure; HDL-C, high-density lipoprotein cholesterol; TG, triglycerides; TC, total cholesterol; HbA1c, hemoglobin A1c; AST, aspartate aminotransferase; ALT, alanine aminotransferase; BUN, blood urea nitrogen; eGFR, estimated glomerular filtration rate; AAC, abdominal aortic calcification; SAAC, severe abdominal aortic calcification.

### 3.2 The association between AIP and AAC

The VIFs for all covariates included in the multivariable regression models were calculated to assess potential collinearity. The VIF values for all variables were below 10, with the highest being 5.8 for waist circumference (**Supplementary Table S1**). This indicates that no significant multicollinearity was present among the selected variables, and all selected covariates were retained for the final analysis. Among all participants, AIP showed a strong positive association with AAC, with each 1% increase in AIP linked to a 31% (OR = 1.31, 95% CI: 1.05–1.64) and 144% (OR = 2.44, 95% CI: 1.38–4.32) increase in AAC risk in unadjusted and fully adjusted models, respectively. Quartile analyses confirmed this trend, with participants in higher AIP quartiles exhibiting significantly elevated AAC risks compared to Q1 (p for trend < 0.003). However, multivariable linear analysis revealed no significant difference between AIP and AAC-24 score or the risk of SAAC after adjusting for covariates (**Table 2**).

**Table 2:** Associations of AIP with AAC score, the risk of AAC and SAAC in different models among all participants.

AIP	Model 1		Model 2		Model 3	
	β/OR (95%CI)	P value	β/OR (95%CI)	P value	β/OR (95%CI)	P value
AAC-24 score						
Per 1 increment	0.35 (-0.01, 0.72)	0.059	0.47 (0.12, 0.82)	0.009	0.36 (-0.15, 0.88)	0.165
Quartile						
Q1	Reference		Reference		Reference	
Q2	0.17 (-0.20, 0.54)	0.375	0.13 (-0.21, 0.47)	0.457	0.14 (-0.20, 0.48)	0.430
Q3	0.35 (-0.02, 0.72)	0.063	0.27 (-0.07, 0.62)	0.117	0.29 (-0.08, 0.66)	0.124
Q4	0.40 (0.03, 0.77)	0.033	0.47 (0.12, 0.82)	0.008	0.33 (-0.11, 0.77)	0.144
P for trend		0.020		0.006		0.102
AAC						
Per 1 increment	1.31 (1.05, 1.64)	0.018	1.45 (1.12, 1.87)	0.004	2.44 (1.38, 4.32)	0.002
Quartile						
Q1	Reference		Reference		Reference	
Q2	1.37 (1.09, 1.74)	0.008	1.41 (1.10, 1.81)	0.008	1.47 (1.12, 1.92)	0.005
Q3	1.51 (1.20, 1.90)	0.001	1.53 (1.19, 1.96)	0.001	1.65 (1.23, 2.21)	0.001
Q4	1.39 (1.10, 1.75)	0.006	1.50 (1.16, 1.95)	0.002	1.63 (1.08, 2.46)	0.019
P for trend		0.004		0.002		0.005
severe AAC						
Per 1 increment	1.28 (0.92, 1.77)	0.142	1.68 (1.14, 2.48)	0.009	1.73 (0.74, 4.05)	0.205
Quartile						
Q1	Reference		Reference		Reference	
Q2	1.08 (0.77, 1.53)	0.653	1.11 (0.76, 1.62)	0.593	1.15 (0.76, 1.74)	0.513
Q3	1.18 (0.84, 1.66)	0.341	1.20 (0.83, 1.75)	0.331	1.30 (0.82, 2.04)	0.262
Q4	1.35 (0.97, 1.88)	0.078	1.69 (1.16, 2.46)	0.006	1.73 (0.92, 3.25)	0.087
P for trend		0.065		0.006		0.102

Model 1: no covariates were adjusted;

Model 2: adjusted for age, gender, race;

Model 3: adjusted for covariates in Model 2 plus education level, marital status, smoking status, alcohol drinking status, waist circumference, BMI, PIR, total cholesterol, triglycerides, hemoglobin A1c, albumin, total bilirubin, AST, ALT, blood urea nitrogen, serum creatinine, eGFR, serum calcium, serum phosphorus, serum uric acid, total 25-hydroxyvitamin D, hypertension, high cholesterol and diabetes status.

AIP, atherogenic index of plasma; AAC, abdominal aortic calcification; BMI, body mass index; PIR, poverty income ratio; AST, aspartate aminotransferase; ALT, alanine aminotransferase; eGFR, estimated glomerular filtration rate; β, effect size; OR, odds ratio CI, confidence interval.

Further sex differences were found in the relationships between AIP and AAC-24 score, the risk of AAC, and SAAC among men and women, as shown in **Tables 3** and **4**. Sex-stratified analyses revealed no significant association between AIP and AAC outcomes in men (**Table 3**). In contrast, women demonstrated a positive association, with AIP linked to higher AAC-24 scores and increased AAC and SAAC risks in both unadjusted and adjusted models (**Table 4**). The AAC-24 score was 1.2 units higher in the unadjusted model (β = 1.20, 95% CI: 0.67, 1.74) and 0.90 units higher in the fully adjusted model (β = 0.90, 95% CI: 0.22, 1.58) for every one-unit (1%) increase in AIP. Additionally, compared with those of Q1, the AAC-24 score of Q2, Q3, and Q4 were 0.41, 0.53, and 0.91 units higher with fully adjusted β (95% CI) values of 0.41 (-0.04, 0.87), 0.53 (0.04, 1.03), and 0.91 (0.31, 1.51), respectively (P for trend = 0.003). Regarding the association between AIP and the risk of AAC, for each unit (1%) increase in AIP in the unadjusted model, AAC risk increased by 95% (OR = 1.95, 95% CI: 1, 40, 2.72) and by 301% in the fully adjusted model (OR = 4.01, 95% CI: 1.65, 9.74). Participants in Q2, Q3, and Q4 of the AIP demonstrated a significant increase in the risk of AAC when compared to those in Q1, with unadjusted ORs (95% CIs) of 1.59 (1.16, 2.17), 1.81 (1.32, 2.48), and 1.95 (1.40, 2.71), (p for trend < 0.001), respectively. In Model 3, this trend persisted to be significant, with Q1 of AIP as the reference. The fully adjusted ORs and 95% CIs of the Q2, Q3, and Q4 categories were 1.61 (1.11, 2.33), 2.01 (1.30, 3.13), and 2.26 (1.16, 4.40), respectively (P for trend = 0.003). Furthermore, we also observed similar results regarding the association between AIP and the risk of SAAC. There was a 143% increase in the risk of SAAC for every unit (1 percent) increase in AIP (OR = 2.43, 95% CI: 1.52, 3.86) in the unadjusted model and in the fully adjusted model by 837% (OR = 9.37, 95% CI: 2.37, 37.03). Participants in Q2, Q3, and Q4 of AIP demonstrated a statistically significant increase in relative risk for developing SAAC when compared to those in Q1, with unadjusted ORs (95% CIs) of 1.85 (1.15, 2.99), 1.80 (1.10, 2.93), and 2.74 (1.69, 4.42), respectively (p for trend < 0.001). This trend remained significant in Model 3. The fully adjusted ORs and 95% CIs of the Q2, Q3, and Q4 categories were 2.10 (1.16, 3.80), 2.48 (1.24, 4.95), and 5.54 (2.05, 14.96), (p for trend = 0.002).

**Table 3:** Associations of AIP with AAC score, the risk of AAC and severe AAC in different models among male.

AIP	Model 1		Model 2		Model 3	
	β/OR (95%CI)	P value	β/OR (95%CI)	P value	β/OR (95%CI)	P value
AAC-24 score						
Per 1 increment	-0.44 (-0.95, 0.08)	0.095	-0.12 (-0.61, 0.37)	0.644	-0.37 (-1.40, 0.65)	0.473
Quartile						
Q1	Reference		Reference		Reference	
Q2	-0.52 (-1.09, 0.06)	0.077	-0.43 (-0.96, 0.10)	0.115	-0.30 (-0.84, 0.24)	0.274
Q3	-0.26 (-0.82, 0.30)	0.359	-0.21 (-0.74, 0.31)	0.427	-0.14 (-0.72, 0.43)	0.632
Q4	-0.54 (-1.08, 0.00)	0.051	-0.27 (-0.79, 0.24)	0.296	-0.37 (-1.12, 0.37)	0.328
P for trend		0.077		0.563		0.482
AAC						
Per 1 increment	0.89 (0.65, 1.22)	0.463	1.04 (0.73, 1.49)	0.812	1.59 (0.73, 3.46)	0.243
Quartile						
Q1	Reference		Reference		Reference	
Q2	1.08 (0.76, 1.54)	0.661	1.16 (0.80, 1.70)	0.437	1.32 (0.88, 1.98)	0.176
Q3	1.14 (0.81, 1.62)	0.445	1.19 (0.82, 1.73)	0.365	1.38 (0.90, 2.11)	0.146
Q4	0.94 (0.67, 1.32)	0.734	1.09 (0.75, 1.58)	0.652	1.24 (0.71, 2.18)	0.457
P for trend		0.694		0.739		0.369
severe AAC						
Per 1 increment	0.69 (0.43, 1.11)	0.123	0.98 (0.56, 1.72)	0.937	0.50 (0.17, 1.50)	0.218
Quartile						
Q1	Reference		Reference		Reference	
Q2	0.54 (0.32, 0.91)	0.021	0.58 (0.33, 1.02)	0.057	0.60 (0.32, 1.11)	0.104
Q3	0.70 (0.44, 1.14)	0.152	0.75 (0.44, 1.29)	0.301	0.73 (0.39, 1.36)	0.320
Q4	0.64 (0.40, 1.02)	0.059	0.86 (0.50, 1.47)	0.587	0.69 (0.30, 1.57)	0.375
P for trend		0.176		0.906		0.412

Model 1: no covariates were adjusted;

Model 2: adjusted for age, race;

Model 3: adjusted for covariates in Model 2 plus education level, marital status, smoking status, alcohol drinking status, waist circumference, BMI, PIR, total cholesterol, triglycerides, hemoglobin A1c, albumin, total bilirubin, AST, ALT, blood urea nitrogen, serum creatinine, eGFR, serum calcium, serum phosphorus, serum uric acid, total 25-hydroxyvitamin D, hypertension, high cholesterol and diabetes status.

AIP, atherogenic index of plasma; AAC, abdominal aortic calcification; BMI, body mass index; PIR, poverty income ratio; AST, aspartate aminotransferase; ALT, alanine aminotransferase; eGFR, estimated glomerular filtration rate; β, effect size; OR, odds ratio CI, confidence interval.

**Table 4:** Associations of AIP with AAC score, the risk of AAC and severe AAC in different models among female.

AIP	Model 1		Model 2		Model 3	
	β (95%CI)	P value	β (95%CI)	P value	β (95%CI)	P value
AAC-24 score						
Per 1 increment	1.20 (0.67, 1.74)	<0.001	1.01 (0.51, 1.50)	<0.001	0.90 (0.22, 1.58)	0.009
Quartile						
Q1	Reference		Reference		Reference	
Q2	0.58 (0.10, 1.07)	0.019	0.44 (-0.00, 0.89)	0.051	0.41 (-0.04, 0.87)	0.072
Q3	0.71 (0.21, 1.21)	0.005	0.53 (0.07, 0.99)	0.024	0.53 (0.04, 1.03)	0.035
Q4	1.26 (0.73, 1.79)	<0.001	1.08 (0.59, 1.56)	<0.001	0.91 (0.31, 1.51)	0.003
P for trend		<0.001		<0.001		0.003
AAC						
Per 1 increment	1.95 (1.40, 2.72)	<0.001	1.98 (1.37, 2.85)	<0.001	4.01 (1.65, 9.74)	0.002
Quartile						
Q1	Reference		Reference		Reference	
Q2	1.59 (1.16, 2.17)	0.004	1.59 (1.13, 2.22)	0.008	1.61 (1.11, 2.33)	0.012
Q3	1.81 (1.32, 2.48)	<0.001	1.82 (1.29, 2.57)	0.001	2.01 (1.30, 3.13)	0.002
Q4	1.95 (1.40, 2.71)	<0.001	1.95 (1.36, 2.79)	<0.001	2.26 (1.16, 4.40)	0.016
P for trend		<0.001		<0.001		0.003
severe AAC						
Per 1 increment	2.43 (1.52, 3.86)	<0.001	2.78 (1.61, 4.80)	<0.001	9.37 (2.37, 37.03)	0.001
Quartile						
Q1	Reference		Reference		Reference	
Q2	1.85 (1.15, 2.99)	0.011	1.83 (1.09, 3.07)	0.023	2.10 (1.16, 3.80)	0.015
Q3	1.80 (1.10, 2.93)	0.019	1.72 (1.01, 2.94)	0.048	2.48 (1.24, 4.95)	0.010
Q4	2.74 (1.69, 4.42)	<0.001	3.01 (1.77, 5.11)	<0.001	5.54 (2.05, 14.96)	0.001
P for trend		<0.001		<0.001		0.002

Model 1: no covariates were adjusted;

Model 2: adjusted for age, race;

Model 3: adjusted for covariates in Model 2 plus education level, marital status, smoking status, alcohol drinking status, waist circumference, BMI, PIR, total cholesterol, triglycerides, hemoglobin A1c, albumin, total bilirubin, AST, ALT, blood urea nitrogen, serum creatinine, eGFR, serum calcium, serum phosphorus, serum uric acid, total 25-hydroxyvitamin D, hypertension, high cholesterol and diabetes status.

AIP, atherogenic index of plasma; AAC, abdominal aortic calcification; BMI, body mass index; PIR, poverty income ratio; AST, aspartate aminotransferase; ALT, alanine aminotransferase; eGFR, estimated glomerular filtration rate; β, effect size; OR, odds ratio CI, confidence interval.

**Figure 2:**
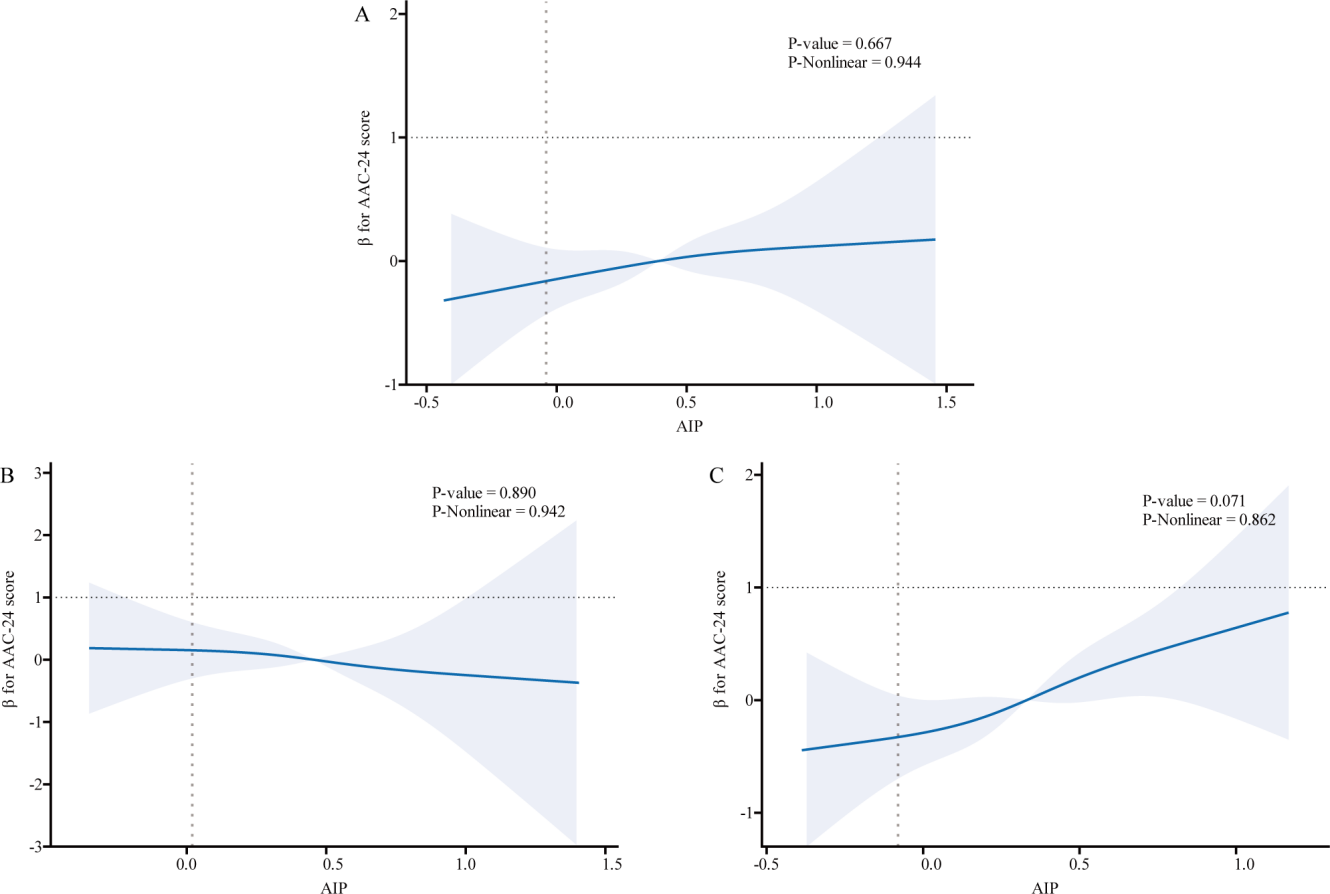
Association relationships between AIP and AAC-24 score. We used restricted cubic splines incorporated in the Cox models with 4 predefined knots at the 15th, 35th, 65th, and 90th centiles to evaluate the association relationships between AIP and AAC-24 score. All models were adjusted for potential confounders in Model 3. The shaded area represents the 95% confidence interval for the predicted values. **(A)** Association between AIP and AAC-24 score among all participants; **(B)** Association between AIP and AAC-24 score among male participants, with the same adjustment for confounders as in panel **(A)**; **(C)** Association between AIP and AAC-24 score among female participants, with the same adjustments. AIP, atherogenic index of plasma; AAC, abdominal aortic calcification.

**Figure 3:**
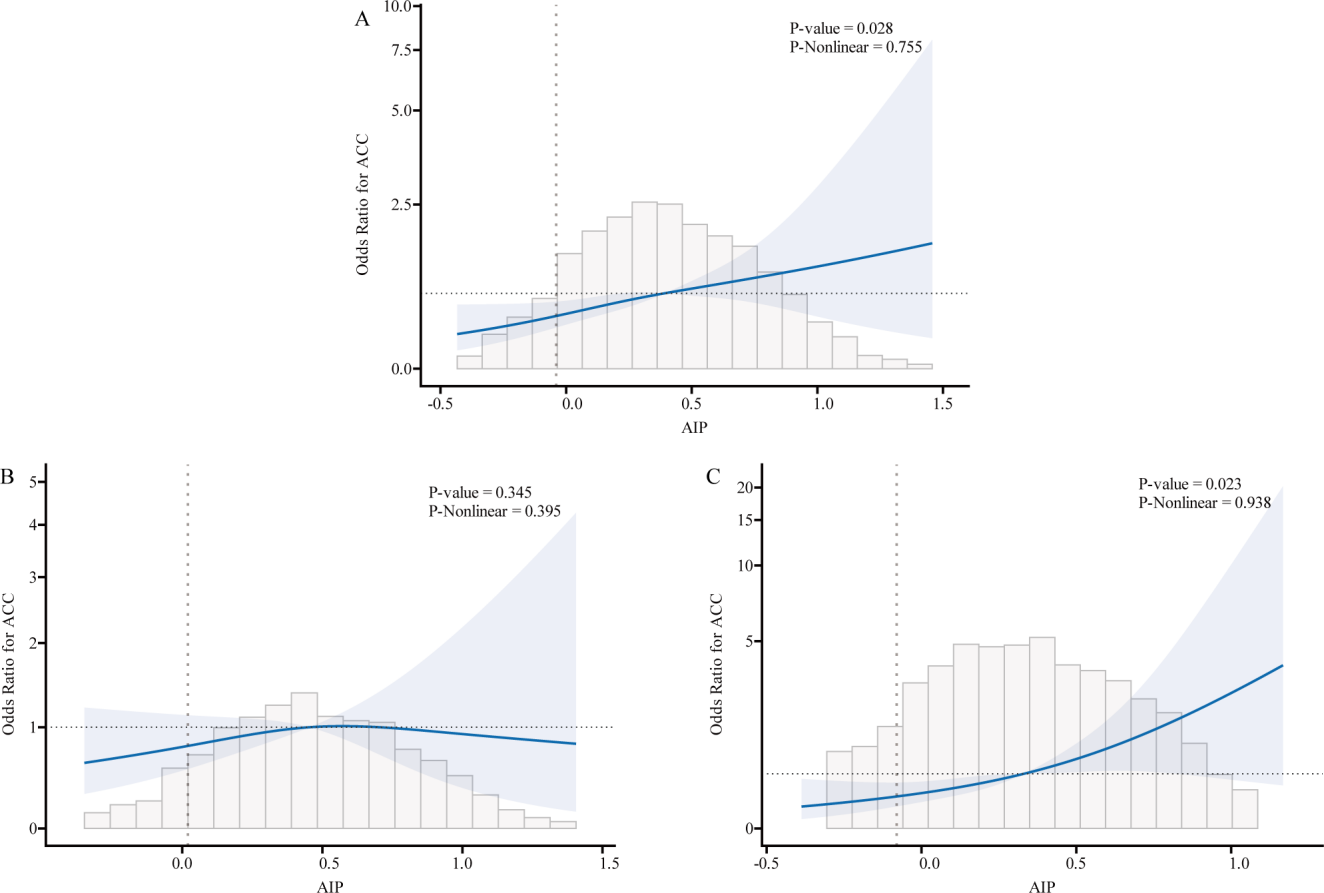
Association relationships between AIP and the risk of AAC. We used restricted cubic splines (RCS) incorporated in the Cox models with 4 predefined knots at the 15th, 35th, 65th, and 90th centiles to evaluate the association relationships between AIP and the risk of AAC. All models were adjusted for potential confounders in Model 3. The shaded area represents the 95% confidence interval for the predicted values. **(A)** Association between AIP and the risk of AAC among all participants; **(B)** Association between AIP and the risk of AAC among male. **(C)** Association between AIP and the risk of AAC among female. AIP, atherogenic index of plasma; AAC, abdominal aortic calcification.

**Figure 4:**
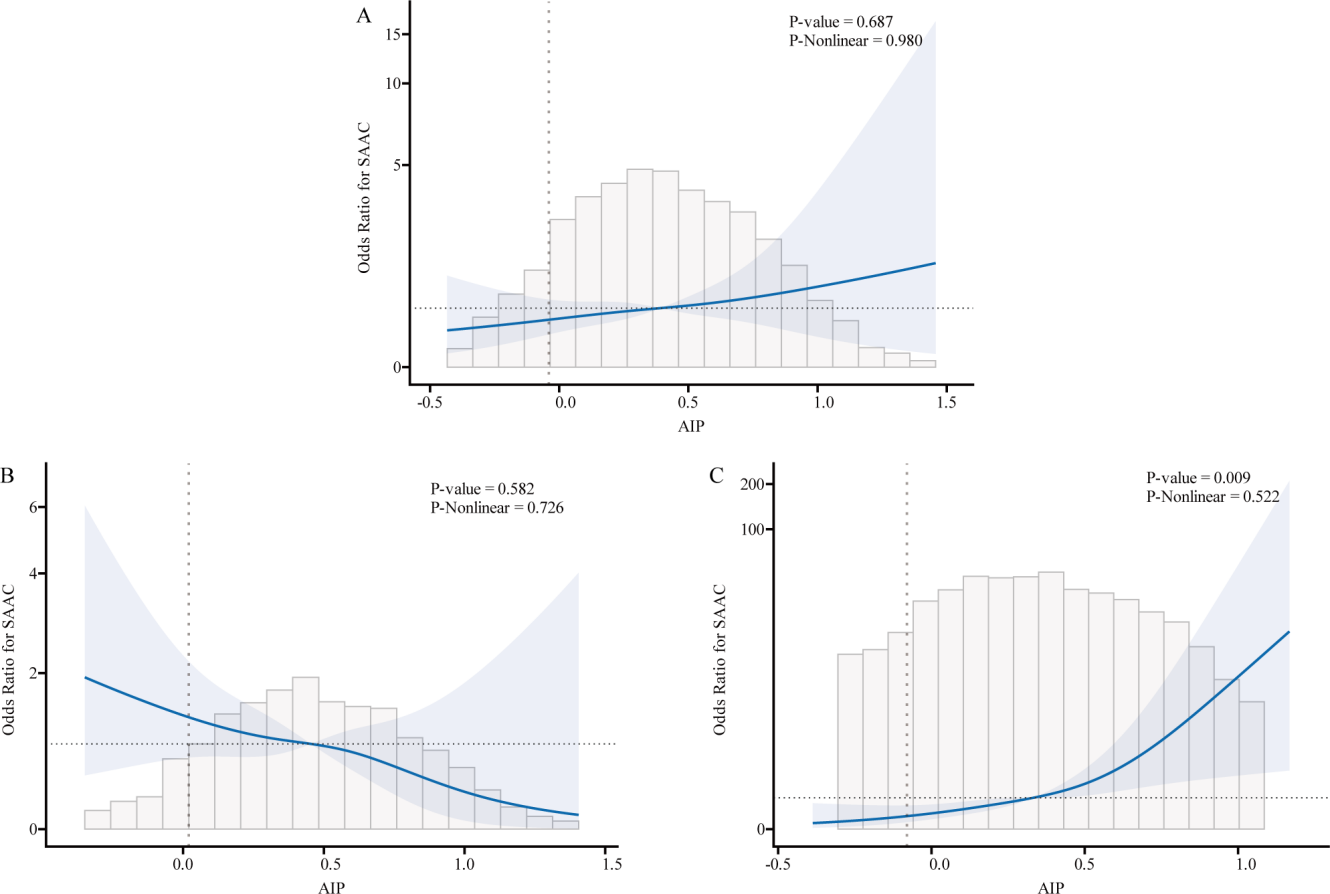
Association relationships between AIP and the risk of SAAC. We used restricted cubic splines (RCS) incorporated in the Cox models with 4 predefined knots at the 15th, 35th, 65th, and 90th centiles to evaluate the association relationships between AIP and the risk of SAAC. All models were adjusted for potential confounders in Model 3. The shaded area represents the 95% confidence interval for the predicted values. **(A)** Association between AIP and the risk of SAAC among all participants; **(B)** Association between AIP and the risk of SAAC among male. **(C)** Association between AIP and the risk of SAAC among female. AIP, atherogenic index of plasma; AAC, abdominal aortic calcification; SAAC, serve abdominal aortic calcification.

We used logistic regression models to perform RCS in order to further investigate the possibility of nonlinearity between the AIP index and AAC. As shown in **Figures 2**–**4**, an RCS model is used for the dose-response analysis. After adjusting for several potential covariates in Model 3, this model demonstrated that there was no significant evidence for a non-linear relationship between AIP and the risk of AAC in all participants (P for nonlinearity = 0.755). This suggests that the association between AIP and AAC can be adequately described using a linear model within the observed data range. Similarly, no evidence of nonlinearity was observed between AIP and AAC outcomes in either sex (P for nonlinearity > 0.5). The P value indicates that no significant evidence supports a non-linear relationship. However, this does not confirm linearity, and further analysis is needed to clarify the association.

### 3.3 Stratified analyses

Significant interactions were observed between AIP and gender (P for interaction = 0.039) and AIP and smoking status (P for interaction = 0.005) for the AAC-24 score. Similarly, interactions between AIP and gender (P for interaction = 0.001) and AIP and smoking status (P for interaction = 0.035) were significant for the risk of severe AAC. These findings suggest potential effect modification by these factors, warranting further investigation. Full results are provided in **Supplementary Figures S1**, **S2**, and **S3**.

### 3.4 Sensitivity analysis

Sensitivity analyses using AAC-8 scores yielded consistent results, confirming the robustness of the findings. Detailed results are provided in **Supplementary Tables S2**-**S4**. Among females, the AAC-8 score increased by 0.33 units for every unit increase in AIP (β=0.33, 95% CI: 0.07, 0.58) in Model 3. Furthermore, compared with that of the lowest quartile, the AAC-8 score increased by 0.32 units for every unit increase in AIP (β=0.32, 95% CI: 0.10, 0.55) in Model 3. There was a 477% higher risk of severe AAC (OR=5.47, 95% CI: 1.36, 21.92) for every unit increase in AIP in Model 3 when defining an AAC-8 score of 3 or higher than SAAC. Furthermore, individuals in the highest quartile of the AIP group had a 338% higher risk of SAAC compared to those in the lowest quartile (OR=3.88, 95% CI: 1.40, 10.74) (P for trend=0.016). Likewise, no difference was found in males, regardless of whether AIP was considered as a continuous variable or as a categorical variable of AAC-8 score (P for trend = 0.671) or the risk of SAAC (AAC-8 score ≥3) (P for trend=0.389).

To further validate the results, we conducted MI. The distribution of baseline characteristics for imputed data is shown in **Supplementary Table S5**. Sensitivity analysis demonstrated consistent results for the association between AIP and AAC-24 score in the fully adjusted model among females (β = 1.02, 95% CI: 0.44, 1.60, P = 0.001), as well as for the risk of AAC (OR = 2.38, 95% CI: 1.24, 4.59, P = 0.009) and SAAC (OR = 6.17, 95% CI = 2.29, 16.65, P < 0.001) (see **Supplementary Table S6**). No significant differences were found among male participants.

## 4 Discussion

This nationally representative cross-sectional survey of 2,811 participants highlights that elevated AIP is associated with an increased risk of AAC, particularly among women. Notably, excessive AIP significantly elevated the AAC-24 score and the risks of AAC and SAAC in women, while no such association was observed in men. Sensitivity analyses confirmed the robustness of these findings. To our knowledge, this is the first study to explore the relationship between AIP and AAC, addressing a critical gap in understanding gender-specific differences in these associations.

AIP, derived from HDL-C and TG, reflects the balance between protective and atherogenic lipoproteins, making it a robust marker of plasma atherogenesis. Studies have demonstrated its predictive value for cardiovascular outcomes, with higher AIP levels associated with increased risks of coronary artery disease, major adverse cardiovascular events, and vascular stiffness. For example, Guo et al. identified AIP as a stronger predictor of coronary artery disease than traditional lipid indices, particularly in postmenopausal women (38), while Kim et al. highlighted its association with adverse cardiovascular events across diverse populations (10). These findings support AIP’s utility in clinical risk stratification for atherosclerosis. Consistent with previous research (39), our study further establishes a linear positive association between AIP and AAC risk, with higher AIP quartiles correlating with significant increases in AAC-24 scores. The highest AIP quartile showed a 63% greater risk of AAC compared to the lowest quartile, particularly in women. This gender-specific difference underscores the influence of physiological and hormonal factors, which warrant further investigation. Furthermore, prior studies did not extensively examine gender differences, underscoring the novelty and unique contribution of our research.

Stratified analyses revealed stronger associations between AIP and AAC in women and interactions with factors such as smoking, BMI ≥ 30, and eGFR < 90. Although these interactions did not reach statistical significance, they suggest potential modifying effects of metabolic and lifestyle factors. For instance, nonsmokers exhibited more pronounced protective effects of lowering AIP on the AAC-24 score and the risk of SAAC (P for interaction = 0.005 and 0.035). In contrast, smokers showed a diminished benefit from lipid control on AAC. This diminished effect may be explained by smoking-induced vascular injury, including inflammation of the endothelial lining, vascular remodeling, and increased arterial stiffness (40). Smoking activates sympathetic nerves through nicotine, leading to increased blood pressure, endothelial dysfunction, and vascular smooth muscle constriction (41). Additionally, smoking impairs nitric oxide levels, which can exacerbate hypertension, diabetes, and atherosclerosis (42). These vascular changes may interfere with the protective role of lipid control on AAC, highlighting the importance of considering smoking status in cardiovascular risk management. Similarly, increased BMI, often associated with IR and metabolic syndrome, may enhance the pro-atherogenic effect of AIP (43). These findings offer new insights into the role of AIP in different populations and clinical subgroups. However, due to the exploratory nature of stratified analyses and the risk of spurious associations from multiple comparisons, the significance of interaction P-values should be interpreted with caution. While adjusting for multiple testing reduces type I errors, it may increase type II errors, potentially masking true associations. Larger, hypothesis-driven cohort studies are needed to validate these interactions and assess their clinical relevance.

The positive correlation between AIP and AAC can be explained by its reflection of lipid metabolism imbalance and its role in promoting vascular calcification (44). AIP integrates TG and HDL-C levels, indicating the balance between atherogenic and protective lipoproteins. Lipid-rich lesions may contain oxidized lipid byproducts, such as dicarboxylic acids, which bind calcium to form insoluble complexes, leading to calcification of aortic smooth muscle cells (45). While calcification enhances plaque stability biochemically, it reduces mechanical stability, increasing the risk of rupture. Elevated levels of oxidized LDL further contribute by promoting smooth muscle cell migration and foam cell formation, exacerbating atherosclerosis (45). LDL particles (LDL-P), particularly small dense LDL (sdLDL), are key drivers of atherosclerosis due to their increased penetration into the vascular endothelium and macrophage uptake (46–48). AIP serves as a reliable surrogate for sdLDL, reflecting lipoprotein atherogenicity and addressing limitations in sdLDL detection methods (6). Moreover, HDL-C in AIP exhibits anti-atherogenic properties, including promoting cholesterol efflux, reducing LDL oxidation, and inhibiting smooth muscle migration and platelet aggregation (49, 50). IR also links AIP with AAC. Elevated lipids contribute to IR via inflammation and lipotoxicity (51), driving metabolic disorders like diabetes and hypertension that accelerate vascular calcification (52). Our findings support this, showing associations between higher AIP levels and markers of metabolic syndrome, including increased BMI, waist circumference, TG, and prevalence of diabetes and hypertension. These pathways highlight the central role of lipid metabolism and IR in the AIP-AAC relationship.

This study highlights a stronger association between AIP and AAC in females, which may be influenced by estrogen deficiency, although other factors also play significant roles. Gender differences in lifestyle factors, such as diet, physical activity, smoking, and alcohol consumption, could contribute to this relationship. Women may have distinct dietary patterns and exercise habits (53), which can influence lipid profiles, while smoking (54) may have a more pronounced negative effect on female cardiovascular health, elevating AIP levels and increasing the risk of AAC. Moreover, metabolic factors, particularly in postmenopausal women, are crucial. These women are more susceptible to metabolic syndrome, including IR, hyperglycemia, and dyslipidemia (55, 56). These factors can accelerate the development of atherosclerosis and enhance the association between AIP and AAC. Age-related changes in fat distribution and metabolism also modify the role of AIP in vascular health (57). Furthermore, women generally have a higher body fat percentage and different fat distribution patterns, such as a greater proportion of abdominal fat, which may further contribute to gender differences in AIP and vascular calcification (58, 59).

The absence of a significant association between AIP and AAC in men could be attributed to inherent differences in lipid metabolism. Men typically exhibit higher LDL-C and lower HDL-C levels (60), which may contribute to a more consistent vascular calcification risk, independent of AIP fluctuations. Additionally, androgens regulate vascular smooth muscle and endothelial function differently from estrogen, potentially leading to gender-specific differences in susceptibility (61). These findings are consistent with previous research showing a stronger correlation between atherosclerotic biomarkers and vascular calcification in women, especially in the postmenopausal population (62).

The AIP has shown promise as a key biomarker for predicting CVD, particularly in high-risk populations such as women. Women with elevated AIP levels may benefit from early cardiovascular screening, which could include lipid profile assessments, glucose monitoring, and evaluation of metabolic syndrome markers. For women with high AIP, personalized treatment strategies targeting lipid metabolism and IR should be considered, including lifestyle interventions, such as diet and exercise modifications and pharmacological approaches (statins or insulin-sensitizing drugs). Furthermore, AIP could serve as an additional factor for refining cardiovascular risk scores, enhancing early detection and tailored prevention strategies for CVD in women.

The primary strengths include the use of NHANES data with a stratified sampling design, comprehensive adjustments for confounders, and MI for missing data, enhancing robustness. However, several limitations should be acknowledged. First, as a cross-sectional study, we cannot establish a causal relationship between the AIP and Atherosclerotic AAC. Causality cannot be inferred from the current data, and future prospective longitudinal studies are needed to determine the directionality and causality of these associations over time. Second, our study utilized data from the NHANES survey, which is representative of the U.S. population. As such, the findings may not be generalizable to populations in other regions or countries, particularly those with different demographic and lifestyle characteristics, and further studies in diverse geographical regions are required to assess the applicability of these findings in other settings. Third, the exclusion of participants with missing data on key variables could have introduced selection bias. These excluded individuals may differ in health or demographic characteristics from those included. While a multivariable logistic regression analysis was conducted after excluding participants with missing data, no sensitivity analysis was performed to assess the potential impact of this bias. Future studies should consider performing sensitivity analyses to evaluate the robustness of findings. Fourth, diseases like DM and hypertension were diagnosed using self-reported data from individuals, which may have introduced bias. Finally, although we controlled for several confounding factors, there may be other unmeasured or residual confounders that could influence the observed associations. Further research should consider a broader set of potential confounders to provide a more comprehensive understanding of the relationship between AIP and AAC.

## 5 Conclusions

This study highlights the association between AIP and AAC, particularly in women. AIP shows promise as a marker for AAC risk, especially in high-risk populations. Future research should explore the underlying mechanisms, particularly hormonal and metabolic factors that may drive gender differences in the associations. Furthermore, prospective studies and clinical trials are essential to validate the predictive value of AIP for AAC risk across diverse populations.

## Data Availability

Publicly available datasets were analyzed in this study. This data can be found here: https://www.cdc.gov/nchs/nhanes/.

## Supplementary material

Supplementary Figure 1 Subgroup analysis for the association between AIP and AAC-24 score. Covariates to be adjusted included age, gender, race, education level, marital status, smoking status, alcohol drinking status, waist circumference, BMI, PIR, total cholesterol, triglycerides, hemoglobin A1c, albumin, total bilirubin, AST, ALT, blood urea nitrogen, serum creatinine, eGFR, serum calcium, serum phosphorus, serum uric acid, total 25-hydroxyvitamin D, hypertension, high cholesterol and diabetes status and covariates related to stratification factors were not adjusted. AIP, atherogenic index of plasma; AAC, abdominal aortic calcification; BMI, body mass index; PIR, poverty income ratio; AST, aspartate aminotransferase; ALT, alanine aminotransferase; eGFR, estimated glomerular filtration rate; β, effect size; OR, odds ratio CI, confidence interval.

Supplementary Figure 2 Subgroup analysis for the association between AIP and the risk of AAC. Covariates to be adjusted included age, gender, race, education level, marital status, smoking status, alcohol drinking status, waist circumference, BMI, PIR, total cholesterol, triglycerides, hemoglobin A1c, albumin, total bilirubin, AST, ALT, blood urea nitrogen, serum creatinine, eGFR, serum calcium, serum phosphorus, serum uric acid, total 25-hydroxyvitamin D, hypertension, high cholesterol and diabetes status and covariates related to stratification factors were not adjusted. AIP, atherogenic index of plasma; AAC, abdominal aortic calcification; BMI, body mass index; PIR, poverty income ratio; AST, aspartate aminotransferase; ALT, alanine aminotransferase; eGFR, estimated glomerular filtration rate; β, effect size; OR, odds ratio CI, confidence interval.

Supplementary Figure 3 Subgroup analysis for the association between AIP and the risk of SAAC. Covariates to be adjusted included age, gender, race, education level, marital status, smoking status, alcohol drinking status, waist circumference, BMI, PIR, total cholesterol, triglycerides, hemoglobin A1c, albumin, total bilirubin, AST, ALT, blood urea nitrogen, serum creatinine, eGFR, serum calcium, serum phosphorus, serum uric acid, total 25-hydroxyvitamin D, hypertension, high cholesterol and diabetes status and covariates related to stratification factors were not adjusted. AIP, atherogenic index of plasma; AAC, abdominal aortic calcification; BMI, body mass index; PIR, poverty income ratio; AST, aspartate aminotransferase; ALT, alanine aminotransferase; eGFR, estimated glomerular filtration rate; β, effect size; OR, odds ratio CI, confidence interval.
