# Supplementary figures and images for: Association between the atherogenic index of plasma and abdominal aortic calcification: results from the National Health and Nutrition Examination Survey 2013–2014
Source: Front Endocrinol (Lausanne). 2025 Feb 17;16:1472267. doi: 10.3389/fendo.2025.1472267 (PMC11872722; doi:10.3389/fendo.2025.1472267)

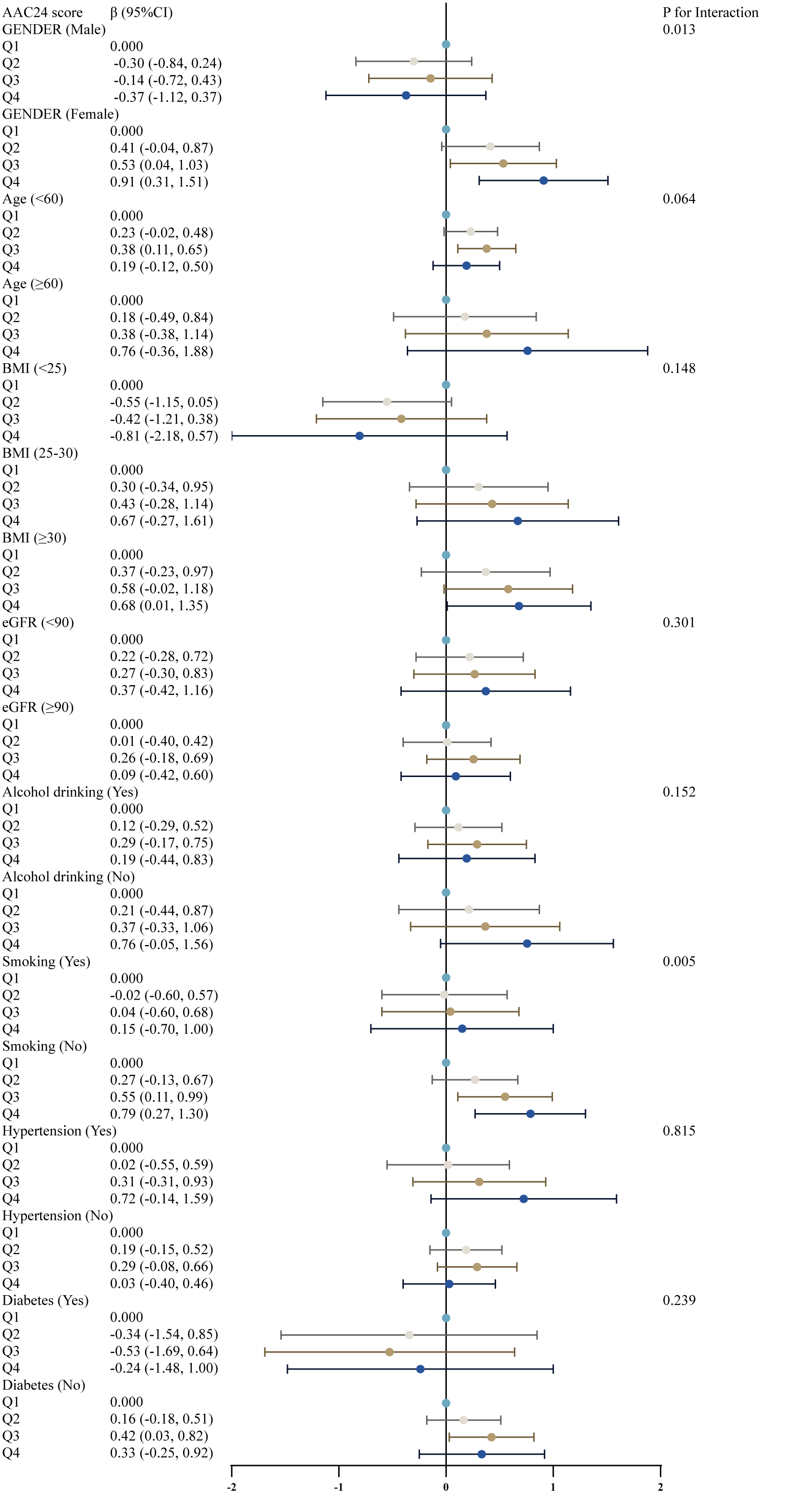

Supplement: Supplementary Figure 1 — Subgroup analysis for the association between AIP and AAC-24 score. Covariates to be adjusted included age, gender, race, education level, marital status, smoking status, alcohol drinking status, waist circumference, BMI, PIR, total cholesterol, triglycerides, hemoglobin A1c, albumin, total bilirubin, AST, ALT, blood urea nitrogen, serum creatinine, eGFR, serum calcium, serum phosphorus, serum uric acid, total 25-hydroxyvitamin D, hypertension, high cholesterol and diabetes status and covariates related to stratification factors were not adjusted. AIP, atherogenic index of plasma; AAC, abdominal aortic calcification; BMI, body mass index; PIR, poverty income ratio; AST, aspartate aminotransferase; ALT, alanine aminotransferase; eGFR, estimated glomerular filtration rate; β, effect size; OR, odds ratio CI, confidence interval. [file Image1.tif]

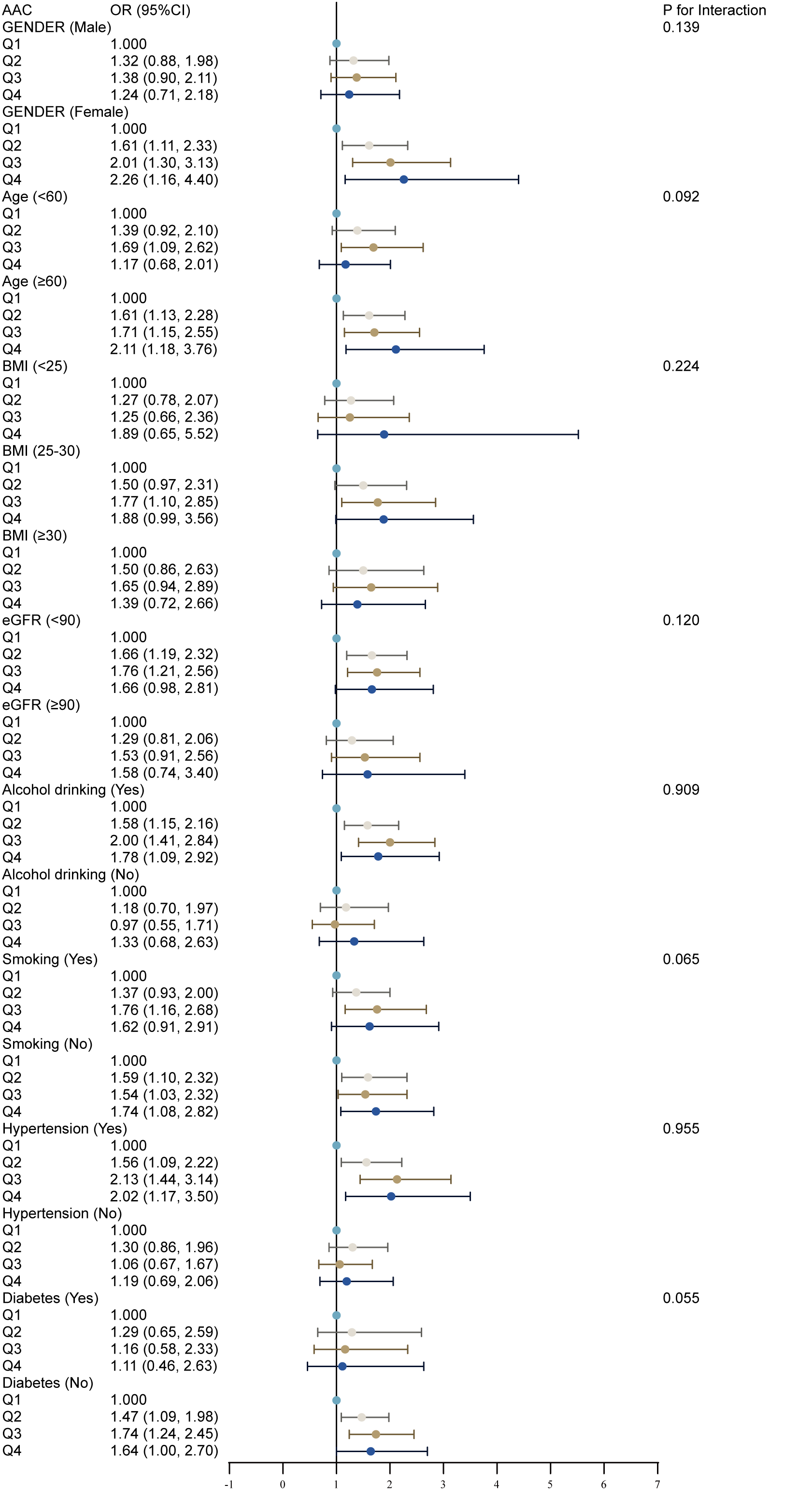

Supplement: Supplementary Figure 2 — Subgroup analysis for the association between AIP and the risk of AAC. Covariates to be adjusted included age, gender, race, education level, marital status, smoking status, alcohol drinking status, waist circumference, BMI, PIR, total cholesterol, triglycerides, hemoglobin A1c, albumin, total bilirubin, AST, ALT, blood urea nitrogen, serum creatinine, eGFR, serum calcium, serum phosphorus, serum uric acid, total 25-hydroxyvitamin D, hypertension, high cholesterol and diabetes status and covariates related to stratification factors were not adjusted. AIP, atherogenic index of plasma; AAC, abdominal aortic calcification; BMI, body mass index; PIR, poverty income ratio; AST, aspartate aminotransferase; ALT, alanine aminotransferase; eGFR, estimated glomerular filtration rate; β, effect size; OR, odds ratio CI, confidence interval. [file Image2.tif]

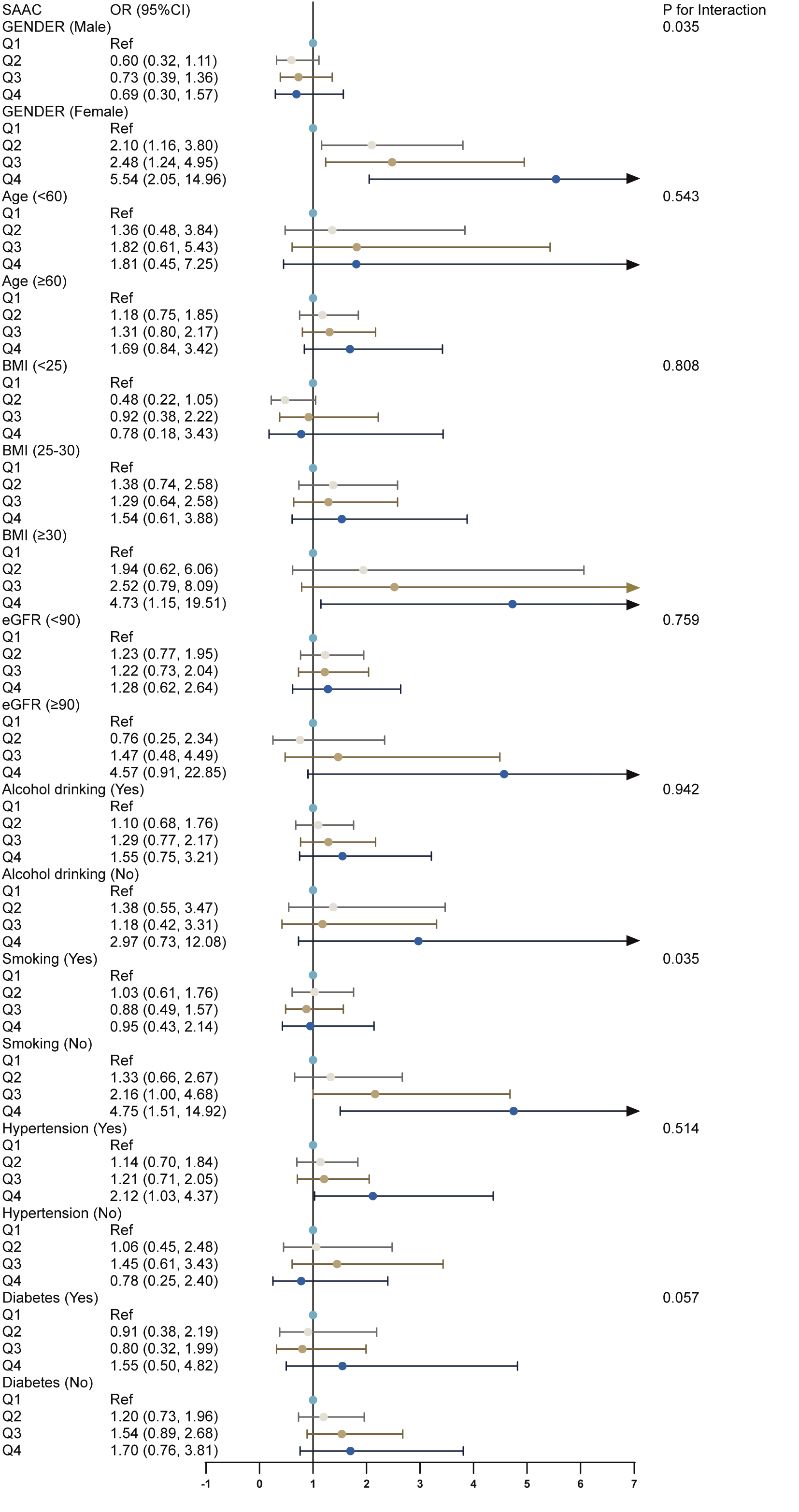

Supplement: Supplementary Figure 3 — Subgroup analysis for the association between AIP and the risk of SAAC. Covariates to be adjusted included age, gender, race, education level, marital status, smoking status, alcohol drinking status, waist circumference, BMI, PIR, total cholesterol, triglycerides, hemoglobin A1c, albumin, total bilirubin, AST, ALT, blood urea nitrogen, serum creatinine, eGFR, serum calcium, serum phosphorus, serum uric acid, total 25-hydroxyvitamin D, hypertension, high cholesterol and diabetes status and covariates related to stratification factors were not adjusted. AIP, atherogenic index of plasma; AAC, abdominal aortic calcification; BMI, body mass index; PIR, poverty income ratio; AST, aspartate aminotransferase; ALT, alanine aminotransferase; eGFR, estimated glomerular filtration rate; β, effect size; OR, odds ratio CI, confidence interval. [file Image3.tif]
