# Supplementary material for: Association between the atherogenic index of plasma and abdominal aortic calcification: results from the National Health and Nutrition Examination Survey 2013–2014
Source: Front Endocrinol (Lausanne). 2025 Feb 17;16:1472267. doi: 10.3389/fendo.2025.1472267 (PMC11872722; doi:10.3389/fendo.2025.1472267)
Supplement: Supplementary file 4 [file Table1.docx]

**Supplementary Table S1** Variance Inflation Factors (VIFs) for covariates in the Multivariable Regression Models

| Variable | VIF |
| --- | --- |
| Age | 2.1 |
| Gender | 1.9 |
| Race | 1.2 |
| Education level | 1.3 |
| Marital status | 1.2 |
| Smoking status | 1.2 |
| Alcohol drinking status | 1.3 |
| Waist circumference | 5.8 |
| BMI | 5.4 |
| PIR | 1.4 |
| Triglycerides | 2.1 |
| Total cholesterol | 1.3 |
| hemoglobin A1c | 1.7 |
| Albumin | 1.5 |
| AST | 3.2 |
| ALT | 3.3 |
| Blood urea nitrogen | 1.8 |
| eGFR | 3.1 |
| Serum uric acid | 1.6 |
| Total 25-hydroxyvitamin D | 1.2 |
| Hypertension | 1.3 |
| High cholesterol | 1.2 |
| Diabetes | 1.6 |
| Serum calcium | 1.4 |
| Serum phosphorus | 1.2 |
| Total bilirubin | 1.1 |
| Serum creatinine | 2.2 |
| AIP | 2.3 |

AIP, atherogenic index of plasma; BMI, body mass index; PIR, poverty income ratio; AST, aspartate aminotransferase; ALT, alanine aminotransferase; eGFR, estimated glomerular ﬁltration rate.

**Supplementary Table S2** Associations of AIP with AAC-8 score and the risk of severe AAC in different models among all participants

| AIP | Model 1 | | Model 2 | | Model 3 | |
| --- | --- | --- | --- | --- | --- | --- |
|  | β/OR (95%CI) | P value | β /OR (95%CI) | P value | β /OR (95%CI) | P value |
| **AAC-8 score** |  |  |  |  |  |  |
| Per 1 increment | 0.15 (0.01, 0.28) | 0.033 | 0.18 (0.05, 0.31) | 0.006 | 0.13 (-0.06, 0.32) | 0.191 |
| Quartile |  |  |  |  |  |  |
| Q1 | Reference |  | Reference |  | Reference |  |
| Q2 | 0.12 (-0.02, 0.25) | 0.092 | 0.10 (-0.02, 0.23) | 0.114 | 0.11 (-0.02, 0.24) | 0.097 |
| Q3 | 0.17 (0.03, 0.30) | 0.015 | 0.14 (0.01, 0.26) | 0.034 | 0.15 (0.01, 0.29) | 0.033 |
| Q4 | 0.18 (0.04, 0.31) | 0.011 | 0.20 (0.07, 0.33) | 0.003 | 0.13 (-0.04, 0.29) | 0.127 |
| P for trend |  | 0.008 |  | 0.003 |  | 0.086 |
| **severe AAC** |  |  |  |  |  |  |
| Per 1 increment | 1.21 (0.85, 1.72) | 0.295 | 1.46 (0.97, 2.20) | 0.070 | 1.82 (0.74, 4.50) | 0.195 |
| Quartile |  |  |  |  |  |  |
| Q1 | Reference |  | Reference |  | Reference |  |
| Q2 | 1.24 (0.86, 1.79) | 0.256 | 1.26 (0.85, 1.87) | 0.251 | 1.33 (0.87, 2.03) | 0.190 |
| Q3 | 1.22 (0.84, 1.76) | 0.300 | 1.20 (0.80, 1.79) | 0.374 | 1.33 (0.82, 2.15) | 0.243 |
| Q4 | 1.30 (0.90, 1.87) | 0.164 | 1.51 (1.01, 2.25) | 0.045 | 1.55 (0.79, 3.03) | 0.199 |
| P for trend |  | 0.203 |  | 0.070 |  | 0.209 |

Model 1: no covariates were adjusted;

Model 2: adjusted for age, gender, race;

Model 3: adjusted for covariates in model 2 plus education level, marital status, smoking status, alcohol drinking status, waist circumference, BMI, PIR, total cholesterol, triglycerides, hemoglobin A1c, albumin, total bilirubin, AST, ALT, blood urea nitrogen, serum creatinine, eGFR, serum calcium, serum phosphorus, serum uric acid, total 25-hydroxyvitamin D, hypertension, high cholesterol and diabetes status.

AIP, atherogenic index of plasma; AAC, abdominal aortic calciﬁcation; BMI, body mass index; PIR, poverty income ratio; AST, aspartate aminotransferase; ALT, alanine aminotransferase; eGFR, estimated glomerular ﬁltration rate; β, effect size; OR, odds ratio CI, conﬁdence interval.

**Supplementary Table S3** Associations of AIP with AAC-8 score and the risk of severe AAC in different models among male

| AIP | Model 1 | | Model 2 | | Model 3 | |
| --- | --- | --- | --- | --- | --- | --- |
|  | β/OR (95%CI) | P value | β /OR (95%CI) | P value | β /OR (95%CI) | P value |
| **AAC-8 score** |  |  |  |  |  |  |
| Per 1 increment | -0.12 (-0.31, 0.06) | 0.198 | -0.03 (-0.21, 0.15) | 0.776 | -0.11 (-0.49, 0.27) | 0.581 |
| Quartile |  |  |  |  |  |  |
| Q1 | Reference |  | Reference |  | Reference |  |
| Q2 | -0.12 (-0.32, 0.09) | 0.268 | -0.09 (-0.29, 0.10) | 0.357 | -0.06 (-0.26, 0.14) | 0.542 |
| Q3 | -0.04 (-0.24, 0.17) | 0.719 | -0.03 (-0.22, 0.16) | 0.759 | -0.01 (-0.22, 0.21) | 0.941 |
| Q4 | -0.14 (-0.33, 0.06) | 0.178 | -0.06 (-0.25, 0.13) | 0.550 | -0.10 (-0.37, 0.18) | 0.487 |
| P for trend |  | 0.310 |  | 0.771 |  | 0.671 |
| **severe AAC** |  |  |  |  |  |  |
| Per 1 increment | 0.69 (0.41, 1.17) | 0.173 | 0.88 (0.48, 1.61) | 0.681 | 0.65 (0.19, 2.17) | 0.480 |
| Quartile |  |  |  |  |  |  |
| Q1 | Reference |  | Reference |  | Reference |  |
| Q2 | 0.60 (0.34, 1.06) | 0.080 | 0.64 (0.35, 1.18) | 0.153 | 0.69 (0.36, 1.31) | 0.255 |
| Q3 | 0.74 (0.44, 1.26) | 0.269 | 0.78 (0.44, 1.37) | 0.383 | 0.73 (0.38, 1.43) | 0.363 |
| Q4 | 0.62 (0.37, 1.05) | 0.074 | 0.76 (0.42, 1.35) | 0.344 | 0.67 (0.27, 1.66) | 0.387 |
| P for trend |  | 0.164 |  | 0.523 |  | 0.389 |

Model 1: no covariates were adjusted;

Model 2: adjusted for age, race;

Model 3: adjusted for covariates in model 2 plus education level, marital status, smoking status, alcohol drinking status, waist circumference, BMI, PIR, total cholesterol, triglycerides, hemoglobin A1c, albumin, total bilirubin, AST, ALT, blood urea nitrogen, serum creatinine, eGFR, serum calcium, serum phosphorus, serum uric acid, total 25-hydroxyvitamin D, hypertension, high cholesterol and diabetes status.

AIP, atherogenic index of plasma; AAC, abdominal aortic calciﬁcation; BMI, body mass index; PIR, poverty income ratio; AST, aspartate aminotransferase; ALT, alanine aminotransferase; eGFR, estimated glomerular ﬁltration rate; β, effect size; OR, odds ratio CI, conﬁdence interval.

**Supplementary Table S4** Associations of AIP with AAC-8 score and the risk of severe AAC in different models among female

| AIP | Model 1 | | Model 2 | | Model 3 | |
| --- | --- | --- | --- | --- | --- | --- |
|  | β (95%CI) | P value | β (95%CI) | P value | β (95%CI) | P value |
| **AAC-8 score** |  |  |  |  |  |  |
| Per 1 increment | 0.44 (0.24, 0.64) | <0.001 | 0.36 (0.18, 0.55) | <0.001 | 0.33 (0.07, 0.58) | 0.013 |
| Quartile |  |  |  |  |  |  |
| Q1 | Reference |  | Reference |  | Reference |  |
| Q2 | 0.26 (0.08, 0.44) | 0.004 | 0.21 (0.04, 0.37) | 0.013 | 0.22 (0.05, 0.39) | 0.012 |
| Q3 | 0.29 (0.11, 0.47) | 0.002 | 0.22 (0.05, 0.39) | 0.011 | 0.24 (0.06, 0.43) | 0.011 |
| Q4 | 0.46 (0.27, 0.66) | <0.001 | 0.39 (0.21, 0.57) | <0.001 | 0.32 (0.10, 0.55) | 0.005 |
| P for trend |  | <0.001 |  | <0.001 |  | 0.004 |
| **severe AAC** |  |  |  |  |  |  |
| Per 1 increment | 2.15 (1.31, 3.52) | 0.002 | 2.23 (1.26, 3.93) | 0.006 | 5.47 (1.36, 21.92) | 0.017 |
| Quartile |  |  |  |  |  |  |
| Q1 | Reference |  | Reference |  | Reference |  |
| Q2 | 2.13 (1.28, 3.52) | 0.004 | 2.06 (1.20, 3.55) | 0.009 | 2.34 (1.28, 4.27) | 0.006 |
| Q3 | 1.81 (1.07, 3.08) | 0.027 | 1.65 (0.93, 2.90) | 0.085 | 2.20 (1.08, 4.50) | 0.031 |
| Q4 | 2.58 (1.54, 4.35) | <0.001 | 2.62 (1.49, 4.60) | 0.001 | 3.88 (1.40, 10.74) | 0.009 |
| P for trend |  | 0.001 |  | 0.004 |  | 0.016 |

Model 1: no covariates were adjusted;

Model 2: adjusted for age, race;

Model 3: adjusted for covariates in model 2 plus education level, marital status, smoking status, alcohol drinking status, waist circumference, BMI, PIR, total cholesterol, triglycerides, hemoglobin A1c, albumin, total bilirubin, AST, ALT, blood urea nitrogen, serum creatinine, eGFR, serum calcium, serum phosphorus, serum uric acid, total 25-hydroxyvitamin D, hypertension, high cholesterol and diabetes status.

AIP, atherogenic index of plasma; AAC, abdominal aortic calciﬁcation; BMI, body mass index; PIR, poverty income ratio; AST, aspartate aminotransferase; ALT, alanine aminotransferase; eGFR, estimated glomerular ﬁltration rate; β, effect size; OR, odds ratio CI, conﬁdence interval.

**Supplementary Table S5** Distributions of variables with complete cases comparing to results from pooling the data with imputed variables from multiple imputation

| Variable^a^ | Complete case | Multiple imputation |
| --- | --- | --- |
| Participants | 2811 | 3015 |
| Age (year) | 57.74 (57.17 ,58.32) | 57.44 (56.87 58.00) |
| Gender (%) |  |  |
| Male | 48.22 | 48.36 |
| Female | 51.78 | 51.64 |
| Race (%) |  |  |
| Mexican American | 6.80 | 6.99 |
| Other hispanic | 4.59 | 4.60 |
| Non-Hispanic White | 72.28 | 71.43 |
| Non-Hispanic Black | 9.68 | 9.77 |
| Other Race | 6.66 | 7.20 |
| Education level (%) |  |  |
| Less than high school | 4.63 | 5.00 |
| High school or equivalent | 31.86 | 10.25 |
| College or above | 63.51 | 84.75 |
| Marital status (%) |  |  |
| Married | 66.02 | 65.71 |
| Unmarried | 33.98 | 34.29 |
| Smoker (%) |  |  |
| Yes | 45.62 | 45.75 |
| No | 54.38 | 54.25 |
| Alcohol user (%) |  |  |
| Yes | 78.13 | 77.62 |
| No | 21.87 | 22.38 |
| Poverty income ratio | 3.15 (2.92, 3.37) | 3.15 (2.94, 3.37) |
| Waist circumference (cm) | 100.01 (99.37, 100.65) | 99.86 (99.20, 100.51) |
| BMI (kg/m^2^) | 28.58 (28.27, 28.89) | 28.54 (28.21, 28.87) |
| SBP (mmHg) | 125.38 (124.30, 126.45) | 125.32 (124.37, 126.27) |
| DBP (mmHg) | 70.68 (69.83, 71.53) | 70.77 (69.88, 71.67) |
| HDL-c (mg/dL) | 54.84 (54.15, 55.54) | 54.72 (54.01, 55.43) |
| TG (mg/dL) | 160.11 (153.77, 166.44) | 160.42 (153.96, 166.88) |
| TC (mg/dL) | 195.48 (194.46, 196.50) | 195.33 (194.23, 196.42) |
| HbA1c (%) | 5.78, (5.73, 5.82) | 5.77 (5.72, 5.82) |
| Total protein (g/dL) | 6.96 (6.91, 7.00) | 6.96 (6.92, 7.01) |
| Albumin (g/dL) | 4.25 (4.23, 4.27) | 4.25 (4.23, 4.27) |
| AST (U/L) | 25.40 (24.35, 26.46) | 25.45 (24.43, 26.48) |
| ALT (U/L) | 24.57 (23.40, 25.74) | 24.74 (23.56, 25.93) |
| BUN (mg/dL) | 14.27 (14.05, 14.50) | 14.23 (14.01, 14.44) |
| Serum calcium (mg/dL) | 9.45 (9.43, 9.48) | 9.45 (9.43, 9.48) |
| Serum phosphorus (mg/dL) | 3.80 (3.76, 3.83) | 3.80 (3.77, 3.83) |
| Serum creatinine (mg/dL) | 0.93 (0.91, 0.95) | 0.93 (0.91, 0.94) |
| eGFR (mL/min/1.73 m^2^) | 82.70 (81.67, 83.72) | 83.11 (82.14, 84.08) |
| Total Bilirubin (mg/dL) | 0.65 (0.63, 0.67) | 0.65 (0.63, 0.67) |
| Serum uric acid (mg/dL) | 5.42 (5.35, 5.49) | 5.41 (5.35, 5.47) |
| Total 25-hydroxyvitamin D (nmol/L) | 75.40 (72.66, 78.14) | 74.98 (72.40, 77.55) |
| Hypertension (%) |  |  |
| Yes | 50.89 | 50.47 |
| No | 49.11 | 49.53 |
| High cholesterol (%) |  |  |
| Yes | 56.61 | 56.13 |
| No | 43.39 | 43.87 |
| Diabetes (%) |  |  |
| Yes | 17.16 | 16.94 |
| No | 82.84 | 83.06 |
| AAC24 score | 1.48 (1.28, 1.69) | 1.48 (1.27, 1.68) |
| AAC (%) |  |  |
| Yes | 29.17 | 28.92 |
| No | 70.83 | 71.08 |
| SAAC (%) |  |  |
| Yes | 9.66 | 9.54 |
| No | 90.34 | 90.46 |

^a^ Data were summarized as mean (95% confidence intervals) or percentage according to their data type.
BMI, body mass index; SBP, systolic blood pressure; DBP, diastolic blood pressure; HDL-C, high-density lipoprotein cholesterol; TG, triglycerides; TC, total cholesterol; HbA1c, hemoglobin A1c; AST, aspartate aminotransferase; ALT, alanine aminotransferase; BUN, blood urea nitrogen; eGFR, estimated glomerular filtration rate; AAC, abdominal aortic calcification; SAAC, severe abdominal aortic calcification.

**Supplementary Table S6** The comparison between complete data analysis and multiple

| Gender | Quartile 1 (β/OR, 95% CI) | Quartile 4 (β/OR, 95% CI), *P* | |
| --- | --- | --- | --- |
|  |  | Complete case | Multiple imputation |
| **AAC-24 score** | |  |  |
| **Overall** |  |  |  |
| Model 3^a^ | 1.0 (Reference) | 0.33 (-0.11, 0.77), 0.144 | 0.37 (-0.05, 0.79), 0.086 |
| **Male** |  |  |  |
| Model 3^b^ | 1.0 (Reference) | -0.37 (-1.12, 0.37), 0.328 | -0.48 (-1.19, 0.23), 0.182 |
| **Female** |  |  |  |
| Model 3^b^ | 1.0 (Reference) | 0.91 (0.31, 1.51), 0.003 | 1.02 (0.44, 1.60), 0.001 |
| **AAC** |  |  |  |
| **Overall** |  |  |  |
| Model 3^a^ | 1.0 (Reference) | 1.63 (1.08, 2.46), 0.019 | 1.63 (1.11, 2.39), 0.013 |
| **Male** |  |  |  |
| Model 3^b^ | 1.0 (Reference) | 1.24 (0.71, 2.18), 0.457 | 1.22 (0.71, 2.09), 0.478 |
| **Female** |  |  |  |
| Model 3^b^ | 1.0 (Reference) | 2.26 (1.16, 4.40), 0.016 | 2.38 (1.24, 4.59), 0.009 |
| **severe AAC** |  |  |  |
| **Overall** |  |  |  |
| Model 3^a^ | 1.0 (Reference) | 1.73 (0.92, 3.25), 0.087 | 1.63 (0.89, 2.98), 0.115 |
| **Male** |  |  |  |
| Model 3^b^ | 1.0 (Reference) | 0.69 (0.30, 1.57), 0.375 | 0.62 (0.28, 1.38), 0.240 |
| **Female** |  |  |  |
| Model 3^b^ | 1.0 (Reference) | 5.54 (2.05, 14.96), 0.001 | 6.17 (2.29, 16.65), <0.001 |

imputation Model 3^a^: adjusted for age, gender, race, education level, marital status, smoking status, alcohol drinking status, waist circumference, BMI, PIR, total cholesterol, triglycerides, hemoglobin A1c, albumin, total bilirubin, AST, ALT, blood urea nitrogen, serum creatinine, eGFR, serum calcium, serum phosphorus, serum uric acid, total 25-hydroxyvitamin D, hypertension, high cholesterol and diabetes status.

Model 3^b:^ adjusted for covariates in Model 3 except for gender.

AIP, atherogenic index of plasma; AAC, abdominal aortic calciﬁcation; BMI, body mass index; PIR, poverty income ratio; AST, aspartate aminotransferase; ALT, alanine aminotransferase; eGFR, estimated glomerular ﬁltration rate; β, effect size; OR, odds ratio CI, conﬁdence interval.
